# Factor Structure and Validation of the Undergraduate Teaching Faculty Investment Questionnaire

**DOI:** 10.3389/fpsyg.2020.593571

**Published:** 2021-01-27

**Authors:** Lizhi Sun, Hong Zhang, Jingjing Xu

**Affiliations:** ^1^College of Education, Qufu Normal University, Qufu, China; ^2^Faculty of Education, East China Normal University, Shanghai, China

**Keywords:** colleges and universities, faculty, undergraduate teaching investment, questionnaire validation, structure

## Abstract

Undergraduate education is very important for higher education. Among the factors affecting the quality of undergraduate education, teaching quality is the most important factor. At present, an important issue that affects teaching quality is insufficient teaching investment. An open-ended questionnaire survey was conducted on 62 faculty members and 65 university students in China. Results found that the undergraduate teaching investment of faculty members mainly consists of four parts: workload investment, ability investment, energy investment, and teaching emotional investment. A preliminary test was conducted on 342 faculty members, and the results of an exploratory factor analysis supported the proposed four-dimensional teaching investment model. The test involved 293 faculty members and confirmatory factor analysis showed that the four-dimensional model had a good degree of fit. A further criterion-related test showed that the four-dimensional teaching investment of faculty members has a significant positive correlation with classroom management, clarity of teaching materials, teacher–student interaction, teaching strategies, and skills application, and work engagement. These results show that the proposed four dimensions of teaching investment effectively measure the teaching investment of faculty members, specifically the indicators of teaching investment and that they promote the development of faculty teaching investment research.

## Introduction

Undergraduate education is important as it has historically been central to the systems of higher education. In recent years, undergraduate education has become a major priority, and there are new attitudes about its importance. In June 2018, the Ministry of Education of China held the New Era National Undergraduate Education Working Conference in Chengdu, Sichuan Province. The conference emphasized that it is important to adhere to the principle of “Taking undergraduate education as the foundation” to promote the “Four Returns,” that is, a return to common sense, a return to duty, a return to original intentions, and a return to the dreams and aspirations of students, emphasizing the need to accelerate the construction of high-level undergraduate education in the new era. The Chinese Education Minister, Chen Baosheng, pointed out that undergraduate education is the root and foundation of universities. Higher education should place undergraduate education at the core of talent cultivation, in a foundational position that connects education and teaching, and at the forefront of the development of education in the new era. Against this background, undergraduate education reform and quality improvement have become important topics in practice and academia in China.

Among the factors affecting the quality of undergraduate education, teaching quality is the most important. In China, the notion that faculty members are “unwilling to teach” has affected teaching quality in higher education. Faculty members are unwilling to invest time and energy in teaching, resulting in insufficient teaching investment. In 2013, the undergraduate audit evaluation plan issued by the Chinese Ministry of Education identified “faculty investment in undergraduate teaching” as an important audit element. Teaching was given only a small or very small priority or emphasis in many faculty members’ recognized value system (Stenstorm, 1991). Among many problems that are related to whether the expected goal of first-class undergraduate education construction can be achieved, the first is poor understanding of the importance of undergraduate education, resulting in the problem of inadequate investment in teacher education in colleges and universities (Lin, 2019). Faculty members in research universities do not have enough teaching time, and less than 40% of faculty members in China were willing to spend their time and energy on prioritizing teaching (Fu, 2017). Another survey showed that the time invested in undergraduate teaching by Chinese faculty members was not lower than that of American research universities (Yan, 2018). This indicates that countries other than China are facing similar problems.

In the 1990s, American researchers revealed that as faculty members were paying more attention to discretionary time, less attention was paid to undergraduate teaching (Massy and Zemsky, 1994). Fifty-four percent of teachers believed that they faced conflicts in teaching, academic research, and administration responsibilities (Eble and McKeachie, 1985), and research activities were paid disproportionately more attention (Anderson and Slade, 2016). The institutions at the bottom of the higher education hierarchy intentionally or unintentionally imitated the top elite universities, leading to a gradual convergence of the organization system and academic behavior, and a decrease in investment in teaching time (DiMaggio and Powell, 1983; Scott, 1995). Faculty members devote more time to scientific research activities and the time investment in teaching activities is decreasing (Milem et al., 2000). The majority of full-time academics in Mexico were more inclined to research (67%). From the perspective of teaching investment, although teaching was an activity in which academics spend more time, compared with the 2008 survey, the investment in teaching has decreased and the average time allocated for research has increased (Estévez-Nenninger et al., 2020). A survey of 13 countries found that, in Canada, the United States, and Hong Kong, faculty members spent more time on research than teaching (Bentley and Kyvik, 2012). Indeed, the analyses of the Chinese data in the CAP and APIKS surveys found that from 2007 to 2018, an obvious change in the faculty members’ work of Chinese research universities was that they paid more and more attention to research and ignored teaching. The ratio of faculty members who prefer teaching and research has changed from 4:6 to 2:8, and the ratio of average teaching time per week to average research time has changed from 1:1.3 to 1:2.4 (Guo and Yao, 2020). The Department of Universities and Science in the United Kingdom published a white paper, “Success as a Knowledge Economy: Teaching Excellence, Social Mobility and Student Choice,” asserting the necessity to reverse the tendency of “emphasizing scientific research and neglecting teaching.”

In China, as early as 1993, the Director of the Higher Education Department of the State Education Commission Zhou (1993) proposed that one of the main reasons for the decline of education quality in colleges and universities in China was an inadequate investment in undergraduate teaching by faculty members. In response to this observation, researchers have begun to study investment in undergraduate teaching, exploring the implications and dimensions of faculty teaching investment.

## Literature Review

### The Connotation and Structure of Faculty Teaching Investment

Ye (1994) pointed out that teaching investment was a kind of teaching attitude or spirit of faculty members, and it was the basic condition of teaching, including pre-class investment, lesson preparation and classroom investment, and teaching. Some scholars also equated the concept of “faculty teaching investment” with “faculty teaching enthusiasm.” Fang and Chen (2010), for example, believed that the faculty teaching enthusiasm was the teaching identity, passion, interest, and corresponding behaviors shown by faculty members in teaching activities. Zhao (2015) similarly defined the faculty teaching investment as three factors: teaching importance, teaching interest, and teaching focus.

Wu and Peng (2017) proposed that the teaching investment of faculty was a positive, active, and voluntary behavior in the process of education and teaching, and it was a dedicated effort to improve the teaching level and the quality of students’ training, including the explicit investment of time, money, materials, as well as the implicit investment of energy and emotion. Liu (2013) also proposed that the teaching investment was the sum of the time, energy, and emotion invested by faculty members in educational and teaching activities. “Teaching investment” is the combination of the time, energy, and emotion that teachers invest in education, teaching, and professional development. The term “combination” means that the “teaching investment” of faculty members is not a simple addition of time, energy, emotion, and other factors but has the meaning of integration (Liu, 2020).

A concept similar to faculty teaching investment is called teaching commitment. This concept can be directly traced back to the theory of organizational commitment proposed by Kanter (1968) and Mowday et al. (1979). Becker and Riel (1999) defined teaching commitment as the investment in faculty teaching (Becker and Riel, 1999), while Lortie (1975) believed that commitment was the desire and action of personal resources for teaching work in universities and colleges. An Australian report (2003) on faculty commitment in teaching pointed out that in the process of faculty commitment in teaching, the personal value system would be more important, which would determine how faculty members allocate scarce and limited personal resources, such as time and energy. Moreover, teaching commitment was also divided into three dimensions by some scholars, namely, identification, effort, and loyalty. Each dimension here was divided into two parts, one was the involvement in subject teaching and the other was the involvement with students as persons (Tyree, 1996). Generally speaking, on the one hand, most of the researchers explained faculty teaching investment from the perspective of structural dimension; on the other hand, the academic community has not widely recognized and unified understanding of the meaning and scope of faculty teaching investment yet.

### Concept of Work Engagement and Its Scale

A concept related to faculty teaching investment is work engagement. Based on the theory of role identity, Kahn (1992) proposed that work engagement is when “organization members are able to place themselves so fully into their task performances.” One of the characteristics of Kahn’s concept of work engagement was that “work engagement should refer to a psychological connection with the performance of work tasks rather than an attitude toward features of the organization or the job” (Christian et al., 2011). After that, many studies drew on Kahn’s (1992) conceptual foundation (e.g., Ashforth and Humphrey, 1995; Rothbard, 2001; Schaufeli et al., 2002; May et al., 2004; Saks, 2006; Rich et al., 2010). Schaufeli et al. (2002) defined work engagement as “a positive, fulfilling, work-related state of mind that is characterized by vigor, dedication, and absorption.” Rothbard (2001) also defined engagement as the “psychological presence but goes further to state that it involves two critical components: attention and absorption.” Thus, it can be seen that the understanding of engagement is more of an explanation from psychological significance perspective for scholars in the field of work engagement. That is, in the Chinese largest comprehensive dictionary, “Cihai,” the interpretation of “investment” is: “Be in it, put it in, and do one thing wholeheartedly.” However, based on the origin of the problem of insufficient teaching investment of faculty, we believe that one of the interpretations of “investment” in the Oxford English Dictionary is more in line with the meaning of this article, that is, “the act of giving time or effort to a particular task in order to make it successful.” Therefore, the faculty teaching investment can be understood as the faculty members’ contribution to the teaching activities in the teaching process, such as money, effort, time, etc.

Kahn (1990) pointed out that work engagement included three dimensions: physiology, cognition, and emotion. Based on Kahn’s concept of work engagement, the researchers developed different work engagement scales. The most often used instrument to measure engagement is the Utrecht Work Engagement Scale (Britt et al., 2001; Schaufeli et al., 2002; Schaufeli et al., 2006), which includes three subscales: vigor, dedication, and absorption (Bakker et al., 2008) a measurement scale containing six items was developed by Saks (2006). While other scholars (Schaufeli et al., 2006) developed a work engagement scale covering three dimensions, vitality, dedication, and absorption, and simplified the previous 17 items to nine (Schaufeli et al., 2006). Later, Schaufeli et al. (2019) proved that UWES-9 can be shortened to an ultra-short version with only three items without causing any major information loss. Although the views are different, it may be summed up that the content dimensions of work engagement mainly include three aspects: physiology, emotion, and cognition.

Although there is a certain correlation between work engagement and faculty teaching investment, there are obvious differences between the two. On the one hand, the basic meaning of “engagement” in work engagement is not consistent with “investment” in academic teaching investment. On the other hand, the work of faculty members includes teaching, scientific research, and social service, and the teaching work is only a part of academic work in colleges and universities. Moreover, faculty teaching in colleges and universities has its unique content, nature, and characteristics, and faculty teaching investment also has its particularity compared with work engagement. Therefore, the theory of work engagement cannot be applied to the teaching work of faculty members in general. In addition, although there were empirical research methods for faculty undergraduate teaching investment, most of the tools were self-designed questionnaires lacking scientific rigor. There is still lack of evaluation tools at home and abroad that follow psychological measurement standards and have reliability to evaluate undergraduate teaching investment of faculty members. Based on the actual teaching behaviors of faculty members, this study compiles the initial measurement scale and forms the final faculty undergraduate teaching investment scale (FUTIS) on the basis of testing the reliability and validity of this scale. The scale can be used to measure the undergraduate teaching investment of faculty in China and other countries.

## Materials and Methods

### Initial Scale Development

At present, there is no consensus on the definition and content of faculty investment in undergraduate teaching in colleges and universities. In order to understand the performance and characteristics of faculty teaching investment, this study used qualitative research methods to obtain comprehensive information through open-ended surveys first. The “academic questionnaire” and “student questionnaire” of “Open-ended Questionnaire of Faculty Investment in Undergraduate Teaching” were developed, in which the “academic questionnaire” is the main one, supplemented by the “student questionnaire,” The purpose of this survey is twofold: (1) To understand the specific characteristics of faculty teaching investment from the perspective of teachers and students, that is, what aspects of faculty teaching investment are reflected, and what are their characteristics? (2) To understand the factors that affect faculty teaching investment, that is, what are the causes that affect enthusiasms of faculty members for teaching investment? The research hopes that through this work, the theoretical conception of the teaching investment structure in colleges and universities can be summarized, and the basic materials for compiling the questionnaire will be obtained. There are three questions in the open-ended questionnaire, for example: “Please list the teaching performance and characteristics of teachers with low undergraduate teaching investment in your opinion, or the teaching performance and characteristics of teachers with high teaching investment (please list as many as you can).” The student open questionnaire also includes three questions, for example: “Please list the performance and characteristics of teachers who have low or high investment in undergraduate teaching in your opinion (please list as many as possible).” “How do you think high or low investment of faculty members in undergraduate teaching will affect you?”

### Participants

The samples of the open-ended survey include two groups: faculty members and students. Among them, 62 faculty members with different teaching ages, professional ranks and titles, college levels and types came from Shandong, Shanghai, Henan, Sichuan, Hubei, Guangxi, and other provinces. A total of 56 valid questionnaires were collected. As the direct object of teaching and the persons who experienced the teaching process, students can sense the degree of faculty teaching investment, so it is necessary to examine the characterization of teaching investment from the perspective of students. A sample of 65 students from QN University (Anonymization) covers humanities, social sciences, and science and engineering.

### Item Selection

Through the collation for the collected open-ended questionnaire, 78 of initial description items of the performance and characteristics on faculty undergraduate teaching investment are obtained. To summarize and analyze these items and refer to the related theories of faculty undergraduate teaching investment and work engagement, this research puts forward four dimensions of the structure of faculty teaching investment, teaching workload, teaching ability, teaching energy, and teaching emotion, and further develops the items to test each dimension. Teaching workload investment refers to the time spent by faculty members on teaching, including the number of courses the teacher teaches, and the time spent preparing lessons, answering questions, and marking homework. Topics include: “I spent more time on scientific research last year,” “I taught 16 lessons per week and above on average in the last semester,” etc. Teaching ability investment refers to the faculty teaching level and the comprehensive quality reflected in the completion of the teaching work, including topics such as “I can make the boring teaching content attractive,” “I can explain the teaching content thoroughly.” Teaching energy investment refers to the mental strength of faculty members in teaching, the degree of hard work, including topics such as “My teaching materials have not been updated for 3 years,” “I carefully design student homework.” Teaching emotional investment refers to attitudes and feelings of faculty members toward teaching, with topics such as: “I am passionate about teaching,” “Teaching is my own job and is the basis of my profession,” and other topics. Experts in pedagogy were invited to analyze the items repeatedly through the frequency calculation of each item, and finally, 49 high-frequency items were put to use. After completing the initial items, three pedagogical faculty members and two psychological faculty members were invited to evaluate the validity of the initial questionnaire mainly accompanied by a Likert 5-point scale (where 1 = valid, 2 = somewhat valid, 3 = general, 4 = somewhat invalid, 5 = invalid). Through the statistics of the evaluation results, the data showed that the evaluation results of experts had significant consistency (*W* = 0.484, *p* < 0.001). Eventually, 12 out of 49 items were deleted, and the remaining 38 items were selected as alternative items after deleting the items where two or more of the faculty members choose “general,” “somewhat invalid,” and “invalid.” Three faculty members in Chinese linguistics, English linguistics, and pedagogy were also invited to discuss alternative items and modify the items with semantic repetition, poor pertinence, difficult understanding, and unclear or inappropriate expression. A total of six items were deleted, and 32 remaining items were used to form the initial questionnaire. The questionnaire was assessed with a Likert 5-point scale anchored at 1 (completely inconsistent) and 5 (completely consistent). The higher the score, the higher the level of teaching investment was for undergraduate faculty in colleges and universities.

### Further Scale Development

#### Participants

This study collected data twice; the initial data were named data set A, and the formal data were called data set B. In the initial data collection, the participants were from Shandong, Hunan, Beijing, Sichuan, Chongqing, Henan, Hubei, Guangdong, Guangxi, Hebei, Shaanxi, Shanghai, Heilongjiang, Shanxi, Inner Mongolia, Zhejiang, Hong Kong, and other provinces or autonomous regions. A total of 342 questionnaires were collected, and 338 valid questionnaires were obtained in data set A after deleting four invalid questionnaires. The participants (123 male faculty and 215 female faculty) included 184 liberal arts faculty and 154 science and engineering faculty; 173 lecturers and below, 119 associate professors, and 46 professors; 20 faculty of “double first-class” universities, 246 of provincial key universities, and 76 of other undergraduate colleges and universities; 131 teaching ages less than 10 years, 128 between 11 and 20 years, and 79 over 20 years.

In the formal data collection, 293 questionnaires were collected from 22 provinces or municipality, including Shandong, Beijing, Gansu, Qinghai, Hunan, Guangdong, Jilin, Zhejiang, Chongqing, Hebei, Anhui, Shanxi, Heilongjiang, Guangxi, Shaanxi, Jiangxi, Hubei, Sichuan, Shanghai, Liaoning, Jiangsu, and Ningxia. After 15 invalid questionnaires were deleted, 278 valid questionnaires were left in data set B. The participants (128 male and 150 female faculty) included 151 liberal arts faculty and 127 science and engineering faculty; 33 faculty with junior and below professional titles, 120 intermediate titles, 89 deputy senior titles, and 36 senior titles; 41 faculty of national “double first-class” universities, 36 of provincial/municipal “double first-class” universities, 138 of provincial key universities, and 63 of other undergraduate colleges and universities; 104 teaching ages below 10 years, 123 between 11 and 20 years, and 51 over 20 years.

#### Analysis Procedure

In order to solve the research questions, we conducted empirical analysis using SPSS (version 22.0) and Amos (version 22.0) statistical software. Firstly, an exploratory factor analysis (EFA) was carried out on 32 reserved items using data set A *via* SPSS 22.0. Secondly, the confirmatory factor analysis was carried out on the data set B using Amos 22.0. Meanwhile, this study performed the convergent validity test by SPSS 22.0.

In the present study, the convergent validity of the “Faculty Teaching Efficacy Scale” was also examined through examining the correlation coefficients among dimensions of FUTIS and teacher’s teaching efficacy and work engagement. Self-efficacy of faculty members has a certain predictive effect on work engagement, and the former is positively correlated with the latter. In this study, the “Utrecht Work Engagement Scale” (UWES-9) developed by Schaufeli et al. (2006) and the “Academic Teaching Efficacy Scale” adapted by the author were used to examine the validity of the questionnaire. The “Academic Teaching Efficacy Scale” was adapted from the “Physical Education Academic Teaching Efficacy Scale” developed by Ma (2005). His scale was divided into four dimensions: classroom management, clarity of teaching materials, interactions between faculty and students, application of teaching strategies and skills, which is suitable for faculty members who engage in various disciplines. The internal consistency coefficients of each dimension in this study were 0.94, 0.95, 0.89, and 0.92, respectively, and the internal consistency coefficient of the total scale was 0.97. So, the adapted questionnaire has good internal consistency. The total score of the work engagement scale can be used to indicate the level of faculty work engagement. The total reliability of the questionnaire on “faculty work engagement” is 0.93. Therefore, this study used four dimensions of the “Faculty Teaching Efficacy Scale” and total score of the “Work Engagement Scale” to test the validity of the questionnaire.

## Results

### Exploratory Factor Analysis

Exploratory factor analysis were carried out on 32 reserved items using data set A. Bartlett’s Test of Sphericity indicated χ^2^= 6,175.49, *p* < 0.001, and the Kaiser–Meyer–Olkin Measure of Sampling Adequacy (KMO) gave values of 0.937, indicating that the data are suitable for factor analyses. According to the theoretical model, the number of fixed factors was four. The results showed that the four-factor model can explain 55.15% of the total item variation, and then five items, Q2, Q5, Q6, Q26, and Q28, were deleted with a degree of commonality less than 0.3. Further, the basis for deletion was that double loading is above 0.3, loading difference is less than 0.3, and loading item is less than 0.4 0.4 (Wang and Jiao, 2017). From this, four factors were finally extracted to explain 69.23% of the total item variation after deleting 13 items, Q8, Q9, Q10, Q12, Q15, Q16, Q17, Q18, Q19, Q22, Q25, Q27, and Q29. For the four-factor model, loadings ranged from 0.74 to 0.83 for Factor 1, which was named undergraduate teaching ability investment, loadings ranged from 0.62 to 0.80 for Factor 2 (undergraduate teaching energy investment), loadings ranged from 0.74 to 0.81 for Factor 3 (undergraduate teaching emotion investment), and loadings ranged from 0.69 to 0.84 for Factor 4 (undergraduate teaching workload investment). And the Internal consistency alphas for the four factors were 0.87, 0.83, 0.79, and 0.70, respectively. The items and loadings contained in each factor were shown in Table 1.

**TABLE 1:** Item loadings of faculty undergraduate teaching investment.

Item	F1	F2	F3	F4
23. I have a lively classroom atmosphere.	0.83			
21. I can make boring teaching content attractive.	0.83			
24. I have a strong ability to control the classroom.	0.82			
20. My “student evaluation” score is ideal.	0.74			
11. I design my student homework carefully.		0.80		
13. I carry on the teaching reflection after class.		0.78		
14. I pay attention to the study and application of new teaching theories and information technology.		0.76		
7. I update the teaching content in time.		0.62		
32. If the lecture is not ideal, I will feel sorry.			0.81	
31. Teaching is my duty and the foundation of my career.			0.77	
30. I am willing to teach everything I have learned to my students.			0.74	
3. I taught an average of 16 classes a week and above last semester.				0.84
4. I taught three or more undergraduate courses last semester.				0.82
1. My teaching workload is well above that required for academic appraisal, promotion, or assessment.				0.69
Contribution rate (%)	38.50	13.61	9.67	7.45

*Based on the information obtained from the existing research and open-ended questionnaire, three periods, 16 periods and one course and three courses are selected as nodes initially. Two items are deleted and the third, fourth items are retained after analyzing by exploratory factor analyses (EFAs).*

### Confirmatory Factor Analysis

**FIGURE 1:**
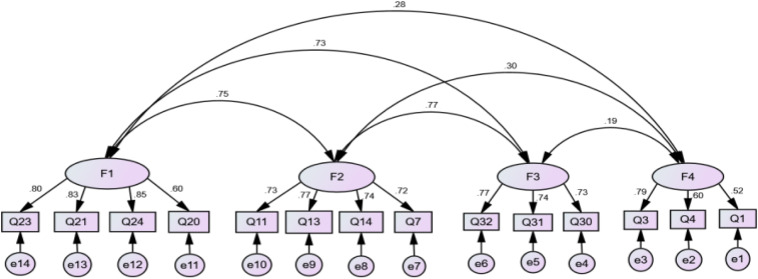
Model of faculty undergraduate teaching investment.

Confirmatory factor analysis and model fitting test were carried out on data set B by using Amos 22.0 in order to confirm the structure of questionnaire model obtained by EFAs. The fitting indexes of the model, χ^2^= 133.59, *df* = 71, χ^2^/*df* = 1.88, Normed Fit Index (NFI) = 0.92, Incremental Fit Index (IFI) = 0.96, Tucker–Lewis Index (TLI) = 0.95, Comparative Fit Index (CFI) = 0.96, root mean square error of approximation (RMSEA) = 0.06, indicated that the structure model fitting of the questionnaire is in good condition. The model was shown in Figure 1.

### Convergent Validity

The convergent validity of the questionnaire was examined by measuring two variables (teaching efficacy and work engagement). The results showed that the correlation coefficients between the four dimensions of the questionnaire and work investment were 0.16–0.75, and the value was the same between the four dimensions of the questionnaire and the dimensions of teaching efficacy in the criterion-related validity, reaching a significant level. Specifically, the dimensions of faculty teaching efficacy have a significant positive correlation with teaching ability investment, teaching energy investment, and teaching emotion investment, which indicated that the higher the teaching efficacy, the higher the investment in teaching ability, energy, and emotion. In addition, there was a low positive correlation between the dimensions of teaching efficacy and teaching workload investment, which indicated that compared with the other three dimensions, the relationship between teaching workload investment and teaching efficacy was less strong. As far as the relationship between the total score of work engagement and faculty teaching investment was concerned, there was a moderate positive correlation between work engagement and teaching ability, energy, and emotion, which indicated that the level of faculty work engagement not only was closely related to their teaching ability, energy, and emotion but also can promote each other to a certain extent. The investment of teaching workload was not correlated with work engagement. The results were shown in Table 2.

**TABLE 2:** The correlation coefficient between the dimensions of the questionnaire and the dimensions of work engagement and teaching efficacy.

	Scale								
	**TAI**	**TENI**	**TEMI**	**TWI**	**CM**	**CTM**	**IBTS**	**ATSS**	**WE**
TAI	1	0.64***	0.61***	0.24***	0.71***	0.75***	0.68***	0.63***	0.57***
TENI		1	0.62***	0.24***	0.59***	0.68***	0.65***	0.58***	0.57***
TEMI			1	0.18**	0.52***	0.60***	0.60***	0.62***	0.50***
TWI				1	0.23***	0.29***	0.26***	0.16**	0.16**

***p < 0.001, **p < 0.01 (two-tailed).

*Faculty Investment in Undergraduate Teaching Scale: TAI, Teaching Ability Investment; TENI, Teaching Energy Investment; TEMI, Teaching Emotion Investment; TWI, Teaching Workload Investment.*

*Teaching Efficacy Scale: CM, Classroom Management; CTM, Clarity of Teaching Materials; IBTS, Interaction Between Teachers and Students; ATSS, Application of Teaching Strategies and Skills.*

*Work Investment Scale: WE, work engagement.*

## Discussion

At present, the evaluation system of “emphasizing scientific research rather than teaching,” which widely exists in colleges and universities, has led faculty members to prefer scientific research (Jia, 2012). Their investment in undergraduate teaching is insufficient, which seriously affects the quality of education and teaching. In order to make faculty members return to their duties, return to teaching, and devote themselves to teaching and education, it is necessary to develop a questionnaire on the undergraduate teaching investment of faculty members so as to provide an objective and scientific understanding of the actual situation of faculty teaching investment in colleges and universities and to provide effective tools for the measurement, adjustment, and improvement of future work.

### Theoretical Implications

The questionnaire prepared by this study included four dimensions: teaching workload investment, teaching ability investment, teaching energy investment, and teaching emotion investment. Specific to the content of each dimension, the investment of teaching workload for faculty members includes the number of class hours per week they work, the number of subjects they taught last semester, and the ratio of workload to the assessment requirements for professional titles. The investment of teaching ability includes the ability of faculty members to control the classroom, to deal with the teaching content, and to mobilize the classroom atmosphere and the ideal degree of students’ evaluation on teaching. Teaching energy investment includes timely updating of teaching content, careful design of students’ homework, after-class teaching reflection, learning and application of new teaching theory and information technology, etc. Faculty members are willing to teach all they have learned to students, regard teaching as the foundation of all, and would feel sad for the unsatisfactory effect of teaching, which can be regarded as teaching emotion investment.

Although research on work engagement by different scholars differs, they all point toward three aspects: the physical, emotional, and cognitive aspects of the individual (Kahn, 1990; Saks, 2006; Schaufeli et al., 2006). Research on the teaching investment of faculty members observed that teaching investment mainly covered three aspects: teaching time, teaching energy, and teaching emotion, which correspond to the three dimensions of work engagement (Liu, 2013; Zhao, 2015). Teaching workload can correspond to faculty teaching time in previous studies. Therefore, the teaching ability investment observed in this study presents a new way of differentiating between existing research.

On the one hand, teaching ability is the basis of faculty effective teaching. The basic characteristics of excellent teaching constructed by Du (2014) were divided into three dimensions: knowledge, ability, and emotion. The teaching effect of faculty members with different teaching abilities varies because of their different participation abilities. On the other hand, the four dimensions of teaching emotion, teaching ability, teaching energy, and teaching time follow a logical thread, asking why invest (teaching emotion), what to invest (teaching ability), and how to invest (teaching energy and teaching time). Thus, teaching investment reflects teaching ability.

The findings of this study help define the implications of faculty teaching investment and will enable future studies to define teaching investment more clearly, enriching teaching theories.

### Practical Implications

Scholars from outside China (Britt et al., 2001; Schaufeli et al., 2002; Saks, 2006) have developed a widely recognized work engagement scale. Scholars within China have further localized this scale, which is now used by the academic community (Zhang and Gan, 2005). However, due to the complexity and particularity of work in colleges and universities, the working engagement scale is not suitable for measuring the teaching work of faculty members. As mentioned above, the work of faculty members includes teaching, scientific research, and social service. In particular, teaching and scientific research as the main work of faculty members have great differences in content, nature, and characteristics. Teaching investment is only one aspect of faculty members’ work investment, and there are differences between teaching and scientific research. Based on this, it is necessary to develop a special questionnaire as a measuring tool for faculty members’ teaching investment in undergraduate teaching.

The development of the “Faculty Teaching Investment Questionnaire” provides a measurement standard for teaching investment of faculty, which can be used in the evaluation of faculty member’s teaching investment, and help improve teaching investment and improve the quality of undergraduate teaching.

### Limitations and Future Directions

The study conducted an open questionnaire survey of 62 faculty members. In addition, 293 faculty members participated in the official questionnaire survey. If these sample sizes are larger, it will be more convincing. Follow-up research will use the compiled “Faculty Teaching Investment Questionnaire” to conduct related research on academic faculty teaching investment to test its effectiveness.

## Conclusion

This study draws the following conclusions. First, university faculty investment in undergraduate teaching is a four-dimensional structure that includes teaching workload investment, teaching ability investment, teaching energy investment, and teaching emotional investment. Second, the indicators of the “Faculty Teaching Investment Questionnaire” meet the requirements of psychological statistics with good reliability and validity and can be used as a tool for further research.

## Data Availability Statement

The original contributions generated for this study are included in the article/Supplementary Material, further inquiries can be directed to the corresponding author.

## Ethics Statement

The studies involving human participants were reviewed and approved by Ethics Committee of Qufu Normal University. The patients/participants provided their written informed consent to participate in this study.

## Author Contributions

LS, HZ, and JX were involved in the project conceptualization, data collection, and analysis. HZ and JX was involved in the presentation of analysis results. All authors were involved in the drafting and revising of the work. All authors provided approval for submission of the contents for publication and agreed to be accountable for the accuracy and integrity of the project.

## Conflict of Interest

The authors declare that the research was conducted in the absence of any commercial or financial relationships that could be construed as a potential conflict of interest.
