# Supplementary material for: Factor Structure and Validation of the Undergraduate Teaching Faculty Investment Questionnaire
Source: Front Psychol. 2021 Jan 27;11:593571. doi: 10.3389/fpsyg.2020.593571 (PMC7873296; doi:10.3389/fpsyg.2020.593571)
Supplement: Supplementary file 1 [file Data_Sheet_1.ZIP › data files 20200809/CFA results.AmosOutput]

CFA results.amw


#### C:\Users\张红\Desktop\data files\CFA results.amw

##### Analysis Summary

##### Date and Time

Date: 2020年8月10日

Time: 11:40:13

##### Title

Cfa results: 2020年8月10日 11:40

##### Groups

##### Group number 1 (Group number 1)

##### Notes for Group (Group number 1)

The model is recursive.

Sample size = 278

##### Variable Summary (Group number 1)

##### Your model contains the following variables (Group number 1)

Observed, endogenous variables

Q23

Q21

Q24

Q20

Q11

Q13

Q14

Q7

Q32

Q31

Q30

Q3

Q4

Q1

Unobserved, exogenous variables

F1

e1

e2

e3

e4

F2

e5

e6

e7

e8

F3

e9

e10

e11

F4

e12

e13

e14

##### Variable counts (Group number 1)

|  |  |
| --- | --- |
| Number of variables in your model: | 32 |
| Number of observed variables: | 14 |
| Number of unobserved variables: | 18 |
| Number of exogenous variables: | 18 |
| Number of endogenous variables: | 14 |

##### Parameter Summary (Group number 1)

|  | Weights | Covariances | Variances | Means | Intercepts | Total |
| --- | --- | --- | --- | --- | --- | --- |
| Fixed | 18 | 0 | 0 | 0 | 0 | 18 |
| Labeled | 0 | 0 | 0 | 0 | 0 | 0 |
| Unlabeled | 10 | 6 | 18 | 0 | 14 | 48 |
| Total | 28 | 6 | 18 | 0 | 14 | 66 |

##### Models

##### Default model (Default model)

##### Notes for Model (Default model)

##### Computation of degrees of freedom (Default model)

|  |  |
| --- | --- |
| Number of distinct sample moments: | 119 |
| Number of distinct parameters to be estimated: | 48 |
| Degrees of freedom (119 - 48): | 71 |

##### Result (Default model)

Minimum was achieved

Chi-square = 133.586

Degrees of freedom = 71

Probability level = .000

##### Group number 1 (Group number 1 - Default model)

##### Estimates (Group number 1 - Default model)

##### Scalar Estimates (Group number 1 - Default model)

##### Maximum Likelihood Estimates

##### Regression Weights: (Group number 1 - Default model)

|  |  |  | Estimate | S.E. | C.R. | P | Label |
| --- | --- | --- | --- | --- | --- | --- | --- |
| Q23 | <--- | F1 | 1.000 |  |
| Q21 | <--- | F1 | 1.106 | .074 | 14.948 | \*\*\* |  |
| Q24 | <--- | F1 | 1.140 | .074 | 15.475 | \*\*\* |  |
| Q20 | <--- | F1 | .852 | .084 | 10.126 | \*\*\* |  |
| Q11 | <--- | F2 | 1.000 |  |
| Q13 | <--- | F2 | 1.012 | .085 | 11.852 | \*\*\* |  |
| Q14 | <--- | F2 | .970 | .085 | 11.394 | \*\*\* |  |
| Q7 | <--- | F2 | 1.018 | .092 | 11.102 | \*\*\* |  |
| Q32 | <--- | F3 | 1.000 |  |
| Q31 | <--- | F3 | 1.099 | .096 | 11.496 | \*\*\* |  |
| Q30 | <--- | F3 | .810 | .072 | 11.249 | \*\*\* |  |
| Q3 | <--- | F4 | 1.000 |  |
| Q4 | <--- | F4 | .834 | .135 | 6.180 | \*\*\* |  |
| Q1 | <--- | F4 | .551 | .093 | 5.910 | \*\*\* |  |

##### Standardized Regression Weights: (Group number 1 - Default model)

|  |  |  | Estimate |
| --- | --- | --- | --- |
| Q23 | <--- | F1 | .805 |
| Q21 | <--- | F1 | .826 |
| Q24 | <--- | F1 | .851 |
| Q20 | <--- | F1 | .598 |
| Q11 | <--- | F2 | .729 |
| Q13 | <--- | F2 | .770 |
| Q14 | <--- | F2 | .738 |
| Q7 | <--- | F2 | .719 |
| Q32 | <--- | F3 | .767 |
| Q31 | <--- | F3 | .743 |
| Q30 | <--- | F3 | .725 |
| Q3 | <--- | F4 | .794 |
| Q4 | <--- | F4 | .600 |
| Q1 | <--- | F4 | .519 |

##### Intercepts: (Group number 1 - Default model)

|  |  |  | Estimate | S.E. | C.R. | P | Label |
| --- | --- | --- | --- | --- | --- | --- | --- |
| Q23 |  |  | 3.964 | .046 | 85.991 | \*\*\* |  |
| Q21 |  |  | 3.946 | .050 | 79.402 | \*\*\* |  |
| Q24 |  |  | 4.014 | .050 | 80.830 | \*\*\* |  |
| Q20 |  |  | 4.011 | .053 | 75.911 | \*\*\* |  |
| Q11 |  |  | 3.989 | .054 | 74.471 | \*\*\* |  |
| Q13 |  |  | 4.119 | .051 | 80.202 | \*\*\* |  |
| Q14 |  |  | 4.058 | .051 | 79.012 | \*\*\* |  |
| Q7 |  |  | 4.061 | .055 | 73.365 | \*\*\* |  |
| Q32 |  |  | 4.392 | .046 | 95.681 | \*\*\* |  |
| Q31 |  |  | 4.306 | .052 | 82.647 | \*\*\* |  |
| Q30 |  |  | 4.590 | .039 | 116.637 | \*\*\* |  |
| Q3 |  |  | 2.673 | .081 | 32.931 | \*\*\* |  |
| Q4 |  |  | 2.633 | .090 | 29.379 | \*\*\* |  |
| Q1 |  |  | 3.773 | .068 | 55.132 | \*\*\* |  |

##### Covariances: (Group number 1 - Default model)

|  |  |  | Estimate | S.E. | C.R. | P | Label |
| --- | --- | --- | --- | --- | --- | --- | --- |
| F4 | <--> | F3 | .122 | .051 | 2.392 | .017 |  |
| F4 | <--> | F2 | .208 | .058 | 3.605 | \*\*\* |  |
| F4 | <--> | F1 | .188 | .053 | 3.567 | \*\*\* |  |
| F2 | <--> | F3 | .295 | .040 | 7.454 | \*\*\* |  |
| F1 | <--> | F3 | .266 | .035 | 7.617 | \*\*\* |  |
| F1 | <--> | F2 | .300 | .039 | 7.602 | \*\*\* |  |

##### Correlations: (Group number 1 - Default model)

|  |  |  | Estimate |
| --- | --- | --- | --- |
| F4 | <--> | F3 | .195 |
| F4 | <--> | F2 | .298 |
| F4 | <--> | F1 | .284 |
| F2 | <--> | F3 | .774 |
| F1 | <--> | F3 | .734 |
| F1 | <--> | F2 | .748 |

##### Variances: (Group number 1 - Default model)

|  |  |  | Estimate | S.E. | C.R. | P | Label |
| --- | --- | --- | --- | --- | --- | --- | --- |
| F1 |  |  | .381 | .049 | 7.777 | \*\*\* |  |
| F2 |  |  | .423 | .064 | 6.641 | \*\*\* |  |
| F3 |  |  | .344 | .049 | 6.949 | \*\*\* |  |
| F4 |  |  | 1.150 | .220 | 5.238 | \*\*\* |  |
| e1 |  |  | .208 | .023 | 9.091 | \*\*\* |  |
| e2 |  |  | .218 | .025 | 8.631 | \*\*\* |  |
| e3 |  |  | .188 | .024 | 7.915 | \*\*\* |  |
| e4 |  |  | .497 | .045 | 10.981 | \*\*\* |  |
| e5 |  |  | .372 | .039 | 9.629 | \*\*\* |  |
| e6 |  |  | .297 | .033 | 9.007 | \*\*\* |  |
| e7 |  |  | .332 | .035 | 9.510 | \*\*\* |  |
| e8 |  |  | .410 | .042 | 9.762 | \*\*\* |  |
| e9 |  |  | .240 | .029 | 8.387 | \*\*\* |  |
| e10 |  |  | .337 | .038 | 8.873 | \*\*\* |  |
| e11 |  |  | .203 | .022 | 9.167 | \*\*\* |  |
| e12 |  |  | .674 | .175 | 3.845 | \*\*\* |  |
| e13 |  |  | 1.424 | .169 | 8.415 | \*\*\* |  |
| e14 |  |  | .949 | .097 | 9.809 | \*\*\* |  |

##### Matrices (Group number 1 - Default model)

##### Total Effects (Group number 1 - Default model)

|  | F3 | F2 | F1 | F4 |
| --- | --- | --- | --- | --- |
| Q1 | .000 | .000 | .000 | .551 |
| Q4 | .000 | .000 | .000 | .834 |
| Q3 | .000 | .000 | .000 | 1.000 |
| Q30 | .810 | .000 | .000 | .000 |
| Q31 | 1.099 | .000 | .000 | .000 |
| Q32 | 1.000 | .000 | .000 | .000 |
| Q7 | .000 | 1.018 | .000 | .000 |
| Q14 | .000 | .970 | .000 | .000 |
| Q13 | .000 | 1.012 | .000 | .000 |
| Q11 | .000 | 1.000 | .000 | .000 |
| Q20 | .000 | .000 | .852 | .000 |
| Q24 | .000 | .000 | 1.140 | .000 |
| Q21 | .000 | .000 | 1.106 | .000 |
| Q23 | .000 | .000 | 1.000 | .000 |

##### Standardized Total Effects (Group number 1 - Default model)

|  | F3 | F2 | F1 | F4 |
| --- | --- | --- | --- | --- |
| Q1 | .000 | .000 | .000 | .519 |
| Q4 | .000 | .000 | .000 | .600 |
| Q3 | .000 | .000 | .000 | .794 |
| Q30 | .725 | .000 | .000 | .000 |
| Q31 | .743 | .000 | .000 | .000 |
| Q32 | .767 | .000 | .000 | .000 |
| Q7 | .000 | .719 | .000 | .000 |
| Q14 | .000 | .738 | .000 | .000 |
| Q13 | .000 | .770 | .000 | .000 |
| Q11 | .000 | .729 | .000 | .000 |
| Q20 | .000 | .000 | .598 | .000 |
| Q24 | .000 | .000 | .851 | .000 |
| Q21 | .000 | .000 | .826 | .000 |
| Q23 | .000 | .000 | .805 | .000 |

##### Direct Effects (Group number 1 - Default model)

|  | F3 | F2 | F1 | F4 |
| --- | --- | --- | --- | --- |
| Q1 | .000 | .000 | .000 | .551 |
| Q4 | .000 | .000 | .000 | .834 |
| Q3 | .000 | .000 | .000 | 1.000 |
| Q30 | .810 | .000 | .000 | .000 |
| Q31 | 1.099 | .000 | .000 | .000 |
| Q32 | 1.000 | .000 | .000 | .000 |
| Q7 | .000 | 1.018 | .000 | .000 |
| Q14 | .000 | .970 | .000 | .000 |
| Q13 | .000 | 1.012 | .000 | .000 |
| Q11 | .000 | 1.000 | .000 | .000 |
| Q20 | .000 | .000 | .852 | .000 |
| Q24 | .000 | .000 | 1.140 | .000 |
| Q21 | .000 | .000 | 1.106 | .000 |
| Q23 | .000 | .000 | 1.000 | .000 |

##### Standardized Direct Effects (Group number 1 - Default model)

|  | F3 | F2 | F1 | F4 |
| --- | --- | --- | --- | --- |
| Q1 | .000 | .000 | .000 | .519 |
| Q4 | .000 | .000 | .000 | .600 |
| Q3 | .000 | .000 | .000 | .794 |
| Q30 | .725 | .000 | .000 | .000 |
| Q31 | .743 | .000 | .000 | .000 |
| Q32 | .767 | .000 | .000 | .000 |
| Q7 | .000 | .719 | .000 | .000 |
| Q14 | .000 | .738 | .000 | .000 |
| Q13 | .000 | .770 | .000 | .000 |
| Q11 | .000 | .729 | .000 | .000 |
| Q20 | .000 | .000 | .598 | .000 |
| Q24 | .000 | .000 | .851 | .000 |
| Q21 | .000 | .000 | .826 | .000 |
| Q23 | .000 | .000 | .805 | .000 |

##### Indirect Effects (Group number 1 - Default model)

|  | F3 | F2 | F1 | F4 |
| --- | --- | --- | --- | --- |
| Q1 | .000 | .000 | .000 | .000 |
| Q4 | .000 | .000 | .000 | .000 |
| Q3 | .000 | .000 | .000 | .000 |
| Q30 | .000 | .000 | .000 | .000 |
| Q31 | .000 | .000 | .000 | .000 |
| Q32 | .000 | .000 | .000 | .000 |
| Q7 | .000 | .000 | .000 | .000 |
| Q14 | .000 | .000 | .000 | .000 |
| Q13 | .000 | .000 | .000 | .000 |
| Q11 | .000 | .000 | .000 | .000 |
| Q20 | .000 | .000 | .000 | .000 |
| Q24 | .000 | .000 | .000 | .000 |
| Q21 | .000 | .000 | .000 | .000 |
| Q23 | .000 | .000 | .000 | .000 |

##### Standardized Indirect Effects (Group number 1 - Default model)

|  | F3 | F2 | F1 | F4 |
| --- | --- | --- | --- | --- |
| Q1 | .000 | .000 | .000 | .000 |
| Q4 | .000 | .000 | .000 | .000 |
| Q3 | .000 | .000 | .000 | .000 |
| Q30 | .000 | .000 | .000 | .000 |
| Q31 | .000 | .000 | .000 | .000 |
| Q32 | .000 | .000 | .000 | .000 |
| Q7 | .000 | .000 | .000 | .000 |
| Q14 | .000 | .000 | .000 | .000 |
| Q13 | .000 | .000 | .000 | .000 |
| Q11 | .000 | .000 | .000 | .000 |
| Q20 | .000 | .000 | .000 | .000 |
| Q24 | .000 | .000 | .000 | .000 |
| Q21 | .000 | .000 | .000 | .000 |
| Q23 | .000 | .000 | .000 | .000 |

##### Minimization History (Default model)

| Iteration |  | Negative eigenvalues | Condition # | Smallest eigenvalue | Diameter | F | NTries | Ratio |
| --- | --- | --- | --- | --- | --- | --- | --- | --- |
| 0 | e | 9 |  | -.524 | 9999.000 | 1797.247 | 0 | 9999.000 |
| 1 | e\* | 4 |  | -.154 | 2.971 | 567.190 | 20 | .449 |
| 2 | e | 1 |  | -.041 | .437 | 368.333 | 6 | .856 |
| 3 | e | 0 | 3107.705 |  | .519 | 218.970 | 5 | .816 |
| 4 | e | 0 | 988.099 |  | .748 | 159.403 | 3 | .000 |
| 5 | e | 0 | 1109.536 |  | .408 | 135.196 | 1 | .971 |
| 6 | e | 0 | 1009.620 |  | .114 | 133.599 | 1 | 1.045 |
| 7 | e | 0 | 1038.723 |  | .006 | 133.586 | 1 | 1.006 |
| 8 | e | 0 | 1059.273 |  | .000 | 133.586 | 1 | 1.000 |

##### Model Fit Summary

##### CMIN

| Model | NPAR | CMIN | DF | P | CMIN/DF |
| --- | --- | --- | --- | --- | --- |
| Default model | 48 | 133.586 | 71 | .000 | 1.881 |
| Saturated model | 119 | .000 | 0 |
| Independence model | 28 | 1736.053 | 91 | .000 | 19.078 |

##### Baseline Comparisons

| Model | NFI Delta1 | RFI rho1 | IFI Delta2 | TLI rho2 | CFI |
| --- | --- | --- | --- | --- | --- |
| Default model | .923 | .901 | .962 | .951 | .962 |
| Saturated model | 1.000 |  | 1.000 |  | 1.000 |
| Independence model | .000 | .000 | .000 | .000 | .000 |

##### Parsimony-Adjusted Measures

| Model | PRATIO | PNFI | PCFI |
| --- | --- | --- | --- |
| Default model | .780 | .720 | .751 |
| Saturated model | .000 | .000 | .000 |
| Independence model | 1.000 | .000 | .000 |

##### NCP

| Model | NCP | LO 90 | HI 90 |
| --- | --- | --- | --- |
| Default model | 62.586 | 33.847 | 99.141 |
| Saturated model | .000 | .000 | .000 |
| Independence model | 1645.053 | 1513.442 | 1784.050 |

##### FMIN

| Model | FMIN | F0 | LO 90 | HI 90 |
| --- | --- | --- | --- | --- |
| Default model | .482 | .226 | .122 | .358 |
| Saturated model | .000 | .000 | .000 | .000 |
| Independence model | 6.267 | 5.939 | 5.464 | 6.441 |

##### RMSEA

| Model | RMSEA | LO 90 | HI 90 | PCLOSE |
| --- | --- | --- | --- | --- |
| Default model | .056 | .041 | .071 | .226 |
| Independence model | .255 | .245 | .266 | .000 |

##### AIC

| Model | AIC | BCC | BIC | CAIC |
| --- | --- | --- | --- | --- |
| Default model | 229.586 | 235.082 |
| Saturated model | 238.000 | 251.626 |
| Independence model | 1792.053 | 1795.259 |

##### ECVI

| Model | ECVI | LO 90 | HI 90 | MECVI |
| --- | --- | --- | --- | --- |
| Default model | .829 | .725 | .961 | .849 |
| Saturated model | .859 | .859 | .859 | .908 |
| Independence model | 6.470 | 5.994 | 6.971 | 6.481 |

##### HOELTER

| Model | HOELTER .05 | HOELTER .01 |
| --- | --- | --- |
| Default model | 191 | 211 |
| Independence model | 19 | 20 |

##### Execution time summary

|  |  |
| --- | --- |
| Minimization: | .011 |
| Miscellaneous: | .200 |
| Bootstrap: | .000 |
| Total: | .211 |
